# 5-hydroxymethylcytosine signature in plasma extracellular vesicle DNA as a diagnostic molecular biomarker for precancerous lesions of gastric cancer

**DOI:** 10.20517/evcna.2025.76

**Published:** 2025-11-19

**Authors:** Haoyu Chen, Tianyu Gao, Hangyu Chen, Lei Zhang, Xianglong Chen, Maimaitiyasen Duolikun, Xiaxuan Li, Xuehui Li, Long Chen, Han Gao, Qi Li, Xinyu Hao, Pingping Zhou, Ningning Ren, Jian Lin, Yangang Wang

**Affiliations:** ^1^School of Graduate, Hebei University of Chinese Medicine, Shijiazhuang 050091, Hebei, China.; ^2^Department of Pharmacy, Peking University Third Hospital, Beijing 100191, China.; ^3^School of Information and Intelligent Engineering, University of Sanya, Sanya 572022, Hainan, China.; ^4^Key Laboratory of Tropical Biological Resources of Ministry of Education, School of Pharmaceutical Sciences, Hainan University, Haikou 570100, Hainan, China.; ^5^School of Information and Communication Engineering, Hainan University, Haikou 570228, Hainan, China.; ^6^Synthetic and Functional Biomolecules Center, Beijing National Laboratory for Molecular Sciences, Peking University, Beijing 100871, China.; ^7^Department of Pharmacy, Peking University Third Hospital Cancer Center, Beijing 100191, China.; ^8^Department of Traditional Chinese Medicine, Peking University Third Hospital, Beijing 100191, China; ^9^Department of Gastroenterology, Hebei Province Hospital of Chinese Medicine, Shijiazhuang 050011, Hebei, China.; ^10^Department of Gastroenterology, Beijing University of Chinese Medicine Third Affiliated Hospital, Beijing 100029, China.; ^#^These authors contributed equally to this work.

**Keywords:** Precancerous lesions of gastric cancer, 5-hydroxymethylcytosine, extracellular vesicle DNAs, molecular biomarker

## Abstract

**Aim:** Precancerous lesions of gastric cancer (PLGC) represent a critical window for prevention. Developing non-invasive tools that can reliably detect these lesions is therefore a prerequisite for lowering gastric-cancer incidence. Recent work has highlighted the diagnostic promise of plasma extracellular vesicle DNAs (evDNAs) and the 5-hydroxymethylcytosine (5hmC)-Seal epigenomic platform. Here we profiled genome-wide 5hmC patterns in circulating evDNA to discover biomarkers and build a classification model.

**Methods:** We performed whole-genome 5hmC-Seal on plasma evDNAs from 67 PLGC patients and 67 healthy individuals. By identifying trend-expressed differentially hydroxymethylated regions (DhMRs), we used machine learning algorithms to screen for diagnostic biomarkers of PLGC and established a corresponding diagnostic model.

**Results:** We ultimately constructed a diagnostic model comprising nine DhMRs. In the test set, the area under the curve (AUC) value was 0.963, with an accuracy of 0.886, sensitivity of 95.45%, and specificity of 81.82%. These results indicate that DhMRs in evDNA can serve as diagnostic biomarkers for PLGC, with good diagnostic capability and reliability. Correlation analysis showed a strong association between the DhMRs in the diagnostic model and clinical pathological indicators of PLGC.

**Conclusion:** We developed a non-invasive diagnostic model for PLGC by profiling 5hmC in plasma evDNA. In both accuracy and inter-batch robustness, it surpasses all previously reported assays. Our findings establish plasma-evDNA 5hmC profiling as a reliable, minimally invasive strategy for the early detection and precise diagnosis of gastric precancerous lesions, and provide a new translational and clinical framework for future work.

## INTRODUCTION

Gastric cancer ranks as the fifth most commonly diagnosed malignancy and is also the fifth leading cause of cancer-related mortality^[1,2]^. Globally, in 2020, there were over 1.08 million new cases reported, resulting in 768,793 deaths^[3]^. Moreover, gastric cancer often presents with few early symptoms, and only 35.63% of gastric cancer cases are diagnosed at early stages (I-II)^[4]^. Studies have shown that in high-risk regions for gastric cancer, such as Japan and South Korea, early screening programs have significantly reduced gastric cancer mortality and improved prognosis, increasing the 5-year patient survival rate to over 54%^[5]^. Additionally, precancerous lesions of gastric cancer (PLGC), including chronic atrophic gastritis (CAG), intestinal metaplasia (IM), and dysplasia (Dys), are the most common and critical pathological conditions of the gastric mucosa that lead to gastric cancer^[6,7]^. Therefore, effective screening for PLGC to advance the treatment timeline and inhibit or reverse the pathological progression of the gastric mucosa is of great significance in reducing the incidence of gastric cancer.

Currently, screening for PLGC primarily relies on gastroscopy. Although advanced techniques such as magnifying endoscopy, chromoendoscopy, narrow-band imaging, autofluorescence, and confocal endomicroscopy can enhance the detection rate of precancerous lesions, diagnosing PLGC accurately remains problematic due to variability in endoscopic imaging and lesion morphology^[8,9]^. Moreover, gastroscopy is expensive and may cause adverse effects such as abdominal discomfort, bleeding, perforation, and infection^[10]^. To date, there is no reliable non-endoscopic biomarker for screening PLGC in the general population. Commonly used serum markers, such as pepsinogen, the pepsinogen I/II ratio, and gastrin-17, are insufficient for effective PLGC screening^[11]^. Additionally, molecular biomarkers identified through sequencing technologies such as microRNA sequencing (microRNA-seq), lipidomics, and proteomics have not yet achieved high levels of accuracy^[12-15]^. Therefore, there is a pressing demand for a low-cost, high-precision, non-invasive or minimally invasive screening tool to detect PLGC and improve diagnostic accuracy.

With the rapid evolution of omics technologies, liquid biopsy - by virtue of its non-invasiveness, minimal discomfort, and logistical convenience - has emerged as a powerful tool for molecular profiling and longitudinal disease monitoring^[16,17]^. To date, blood-based assays for PLGC have chiefly interrogated circulating proteins, RNAs, and metabolites^[18-20]^. While these analytes report on gene expression, functional activity, and metabolic flux, they are intrinsically labile and difficult to preserve^[21,22]^. Cell-free DNA (cfDNA) offers superior stability, and the advent of high-throughput sequencing has uncovered myriad cfDNA- and ctDNA-derived biomarkers that now guide cancer diagnosis and management^[23,24]^. Yet cfDNA is predominantly released during cell death, providing a snapshot of terminal events; in early malignancies, ctDNA is often present at vanishingly low concentrations^[25,26]^. Extracellular vesicle DNAs (evDNAs) are rapidly gaining attention. The lipid bilayer of extracellular vesicles (EVs) shields its molecular cargo from nucleases and proteases, while enabling both short- and long-range intercellular communication^[21,27-29]^. These nanocarriers traverse endothelial barriers, enter the circulation, and deliver a stable, information-rich record of disease status that can be harnessed for diagnosis, therapeutic monitoring, and deep molecular characterization^[30-32]^. Functioning in paracrine and endocrine signaling networks, evDNAs themselves constitute a high-density message stream. Recent studies have sequenced evDNAs directly from plasma to generate robust classifiers for advanced pancreatic and colorectal cancers^[33,34]^. Collectively, these findings position evDNAs as a next-generation biomarker with transformative potential for the early detection and precision management of human disease.

Recent studies have revealed a close link between PLGC and epigenetics, with DNA methylation being identified as a key factor associated with PLGC and serving as a potential diagnostic biomarker. The 5-hydroxymethylcytosine (5hmC) is a crucial intermediate in the epigenetic process from DNA methylation to demethylation and is vital for numerous physiological and pathological processes^[35,36]^. Previous research has shown that 5hmC is highly accurate and sensitive, making it a valuable epigenetic biomarker for various human diseases, including gastric cancer^[37]^, esophageal cancer^[38]^, hepatocellular carcinoma^[39]^, and colorectal cancer^[40]^. The latest findings also indicate that the 5hmC molecular landscape is associated with the development of CAG in the pathological stages of PLGC^[41]^. Thus, detecting 5hmC in blood evDNAs could help elucidate the epigenetic changes in PLGC and identify epigenetic molecular markers with diagnostic potential.

This study utilized plasma evDNAs from patients with PLGC and a matched cohort of healthy individuals with similar gender and age. It also employed the whole-genome 5hmC-Seal technology for evDNAs, which our team developed and optimized for clinical plasma samples. The objective was to clarify the distribution of 5hmC in the PLGC genome, investigate the characteristics of 5hmC-enriched regions, identify genes with differential 5hmC modifications between PLGC and healthy individuals as potential biomarkers, and subsequently develop a diagnostic model to facilitate non-invasive detection of PLGC.

## METHODS

### Design of study and participants

This study adopted a case-control design, recruiting 67 patients with PLGC and matching them with a control group based on age and sex. All participants were from the Hebei Province Hospital of Chinese Medicine between January and December 2024, with the ethics approval number HBZY2023-YS-134-01. The diagnosis of precancerous gastric lesions was based on *the Guidelines for Diagnosis and Treatment of* *Chronic Gastritis in China (2022, Shanghai)*^[42]^ and *the updated Sydney System* for disease classification and grading. Inclusion criteria included: (1) age ranging from 20 to 70 years, regardless of gender; (2) meeting the diagnostic criteria for precancerous gastric lesions, with endoscopy and pathology assessed by at least two relevant experts; (3) informed consent, voluntary participation in the study, and signature of consent form. Exclusion criteria were: (1) patients with severe hepatic and renal impairment, hematologic disorders, autoimmune diseases, endocrine disorders, or other serious primary diseases affecting life expectancy; (2) patients with malignancies, acute infections, or other major illnesses; (3) pregnant, miscarried, or breastfeeding women; (4) patients unable or unwilling to cooperate in the collection of relevant information due to illness or other reasons.

### Sample size calculation

In this study, we assessed the performance of existing diagnostic models for PLGC through a literature review and found that their area under the curve (AUC) values generally ranged from 0.6 to 0.9^[18-20]^. Drawing on our research team’s previous experience in constructing diagnostic models, we selected an AUC of 0.90 as the benchmark for estimating the required sample size. We calculated the sample size using the formula provided by the Power Analysis and Sample Size (PASS) software (2021 edition). The ratio of the positive to negative groups was set at 1:1, and the alternative hypothesis AUC was set at 0.90. Additionally, we designated 0.75 as the null hypothesis value for the test criterion.

**Figure:**
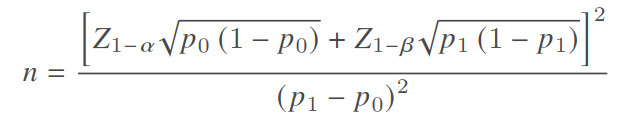


Firstly, we establish the values for α and β at 0.05 and 0.2, respectively. For α, the Z score corresponding to a 95% confidence level, Z_1-α_, is approximately 1.96. For β, assuming a power of 80%, Z_1-β_ is roughly 0.84.

Next, we calculate the variance for the baseline proportion *p*0 = 0.6 and the new proportion *p*1 = 0.9:

**Figure:**
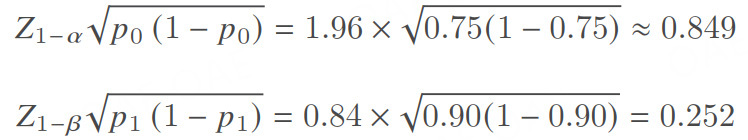


We then sum these products and divide by the difference between *p*1 and *p*0:

**Figure:**
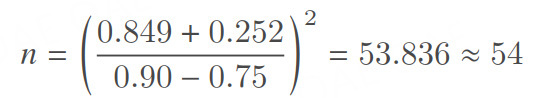


Ultimately, based on the formula, we determined that the minimum required sample size per group was 54 individuals. With this in mind, we endeavored to recruit more than this threshold number of participants during the study period. In the end, we enrolled a total of 67 patients to ensure the reliability and validity of the study results.

### Plasma separation and evDNAs extraction

In line with our previously established experimental protocol^[43]^, we initiated by collecting whole blood samples from patients via standard venipuncture. These samples were stored in cfDNA collection tubes (Roche) at a temperature range of 15 to 25°C. Each sample was processed within 24 h, with plasma separated by centrifugation. EVs were extracted using an exosome extraction kit (H-Wayen Exosome Extraction Kit, EIQ3-02001, China). Briefly, 1 mL plasma was mixed with 20 µL Reagent C, vortexed, and incubated for 15 min at 37 °C, then centrifuged at 10,000 × *g* for 10 min; the supernatant was kept, combined with 250 µL Reagent A, incubated for 30 min on ice, and pelleted at 3,000 × *g* for 1 min. After discarding the supernatant, the pellet was resuspended in 1 mL PBS, 250 µL Reagent B was added, the mixture was incubated for 30 min at 4 °C, and centrifuged again at 3,000 × *g* for 1 min. The final EV-rich pellet was resuspended in 200 µL PBS and stored at -80 °C until use. The extraction and purification of evDNAs were conducted using the Quick-DNA Miniprep Kit (ZYMO), which involved adding BioFluid&Cell buffer, proteinase K, genomic binding buffer, genomic DNA (gDNA) wash buffer, incubation, and centrifugation at 12,000 × *g*. Prior to library construction, quality control was performed using nucleic acid electrophoresis.

### Characterization of EVs

Exosome size and concentration were determined by nanoparticle tracking analysis (NTA) performed at VivaCell Biosciences on a ZetaView PMX 110 instrument (Particle Metrix, Meerbusch, Germany) running ZetaView 8.06.01 software. Isolated exosomes were diluted in 1 × phosphate-buffered saline (PBS, Biological Industries, Israel) to the optimal concentration for measurement. For each sample, videos were captured at 11 consecutive positions and subsequently analyzed. The system was calibrated with 110 nm polystyrene standards, and measurements were carried out at 23-30 °C.

To confirm the size and morphology of patient-derived EVs, samples were prepared for negative-stain transmission electron microscopy (TEM). A 5-μL droplet of EVs suspended in PBS was applied onto a 200-mesh Formvar/carbon-coated copper grid and allowed to adsorb for 1 min. Excess liquid was gently blotted with filter paper, and the grid was stained with four consecutive drops of 1.5% (w/v) uranyl acetate. After brief washing and air-drying, specimens were imaged with an FEI Tecnai G2 Spirit transmission electron microscope (TEM) operated at 60-120 kV.

Purified EVs were lysed on ice in radio-immunoprecipitation assay (RIPA) buffer (Thermo Fisher Scientific) containing 1 × protease inhibitor and 1 mM phenylmethylsulfonyl fluoride (PMSF). After centrifugation at 12,000 × *g* for 10 min at 4 °C, the supernatant was transferred to a fresh tube. Protein concentration was determined with the bicinchoninic acid (BCA) Protein Assay Kit (Thermo Fisher Scientific). Fifty micrograms of protein per sample were separated on 12% SDS-PAGE (sodium dodecyl sulfate-polyacrylamide gel electrophoresis) and electro-transferred to polyvinylidene fluoride membrane (PVDF) membranes. Membranes were blocked with 5% non-fat milk at room temperature, then incubated overnight at 4 °C with primary antibodies against CD9 (ab236630, Abcam), CD63 (67605-1-Ig, Proteintech), CD81 (66866-1-Ig, Proteintech), HSP70 (ab5439, Abcam), TSG101 (28283-1-AP, Proteintech) and Albumin (16475-1-AP, Proteintech). After washing, membranes were probed with horseradish peroxidase (HRP)-conjugated secondary antibodies and visualized using an ECL kit (Thermo Fisher Scientific) on a Tanon chemiluminescence imager (China).

### Construction of 5hmC library and high-throughput sequencing

In this study, the 5hmC libraries for all samples were constructed using an efficient 5hmC sealing technique, consistent with our previous experiments^[44]^. According to the kit protocol, evDNAs isolated from plasma were subjected to end repair and 3’-adenylation using the Hyper Prep Kit (KAPA Biosystems). Illumina-compatible adapters were then ligated for labeling. Subsequently, the connected evDNAs were glycosylated in a prepared solution. Then C_39_H_51_N_5_O_8_S (DBCO-PEG_4_-biotin, Click Chemistry Tools) was added and incubated. Next, the DNA was purified and cleaned. Subsequently, the purified DNA was incubated with streptavidin-coated beads (Life Technologies) in a buffer containing Tween 20, followed by washing and polymerase chain reaction (PCR) amplification. Next, we further purified the PCR products using AMPure XP beads (Beckman). After quantifying the concentration and performing fragment size quality control (QC), paired-end high-throughput sequencing (150 bp) was conducted on libraries meeting the quantitative standards using the NovaSeq 6000 platform. Post-sequencing library QC was performed, including assessments of Q30 score and duplication rate. The peak of DNA fragments was mainly concentrated around 180 bp, with a size range of 0-600 bp. The average Q30 score was 91.2%, and the duplication rate was approximately 39.1%.

### Exploration and alignment of modified regions

The raw sequencing data were first aligned to the human genome using Bowtie2^[45]^, and duplicate reads were removed through filtering with SAMtools^[46]^. Subsequently, the paired-end reads were normalized and compared to the total read count, formatted in BedGraph for initial analysis. These were then converted to bigwig format to facilitate visualization in genome browsers such as the University of California Santa Cruz (UCSC) Genome Browser^[47]^. Potential hydroxymethylated regions (hMRs) were identified using Model-based Analysis of ChIP-seq (MACS; version 2.2.7.1), and overlapping peaks were merged using bedtools merge. Only peaks present in more than 10 samples and smaller than 1,000 bp were retained, while genomic regions known to produce artifact signals were excluded. For each patient, hMRs were generated by intersecting individual peak call files with a combined peak file^[48,49]^. Additionally, intervals overlapping ENCODE blacklist regions, satellite repeats, or sex chromosomes (X/Y) were excluded to minimize copy-number or mapping artefacts.

### Identification of differential hydroxymethylation regions and associated bioinformatics analysis

Differential analysis of 5hmC regions between PLGC and healthy samples was performed using the limma package in R. We identified differential hydroxymethylated regions (DhMRs) using a two-sided Wilcoxon rank-sum test (criteria: |log2 fold change| > 0.5 and *P* value < 0.01 for high-confidence DhMR identification; a relaxed threshold of *p* < 0.05 was used for exploratory clustering visualization) for further analysis^[50]^. Following Correa’s cascade, gastric adenocarcinoma is viewed as a continuous progression from chronic gastritis to cancer. Mfuzz was used to select DhMRs whose 5hmC signals show a monotonic upward or downward trend across successive pathological stages^[51]^. The corresponding DhMGs were matched. The Kyoto Encyclopedia of Genes and Genomes (KEGG) pathway analysis and Gene Ontology (GO) enrichment analysis were performed utilizing the clusterProfiler package in the R programming environment^[52]^.

### Selection of feature regions and construction of diagnostic model

To identify the most informative diagnostic DhMRs from the initial pool, we compared three feature-ranking algorithms - linear discriminant analysis, logistic regression and random forest - implemented in Python 3.8. A marker was advanced to the modelling stage only if it was selected by at least two of the three methods. The training set was then submitted to a stratified 5-fold cross-validation scheme repeated 10 times (50 folds in total) to build a parameter-free ensemble; model performance was finally assessed on the held-out test set. The ensemble itself combined three base learners: a neural network, a random forest and a stochastic-gradient-descent (SGD) classifier. The probability outputs of these learners were concatenated into a meta-feature matrix that fed a support-vector-machine (SVM) combiner for the ultimate class assignment [Supplementary Figure 1]. Specifically, the convolutional layer structure of the neural network was 256*128*64*32*16*8*4*1, with the rectified linear unit (ReLU) activation function and binary cross-entropy loss function. The training duration was set to 200 epochs. The random forest consisted of 300 decision trees. In our stochastic gradient descent model, we employ log-loss with elastic-net regularization, setting the L1-to-L2 ratio to 0.5. This blend of L1 and L2 penalties fosters sparsity while safeguarding model stability. A meta-classifier was trained using the limited-memory Broyden-Fletcher-Goldfarb-Shanno algorithm (L-BFGS) to optimize the Support Vector Machine (SVM) model. The SVM meta-classifier was used for the final prediction, with a classification threshold of 0.5 (i.e., 1 for probabilities greater than 0.5 and 0 for probabilities less than 0.5). We assessed the model’s discriminative performance using the receiver operating characteristic (ROC) curve and its AUC, from which the corresponding specificity and sensitivity were derived.

### Statistical analysis

Continuous variables are presented as mean ± standard deviation and compared among groups using one-way analysis of variance (ANOVA). Categorical variables are summarized as counts or proportions and compared using the chi-square test. For high-dimensional 5hmC-Seal data, differential analysis was performed using the limma-trend framework, with *P*-values adjusted by the Benjamini-Hochberg false discovery rate (FDR). The performance of the diagnostic model was evaluated using the AUC with 95% confidence intervals estimated by 2,000 bootstrap resamplings. All statistical analyses were conducted using R software.

## RESULTS

### Demographics and clinical characteristics of the study population

**Figure 1:**
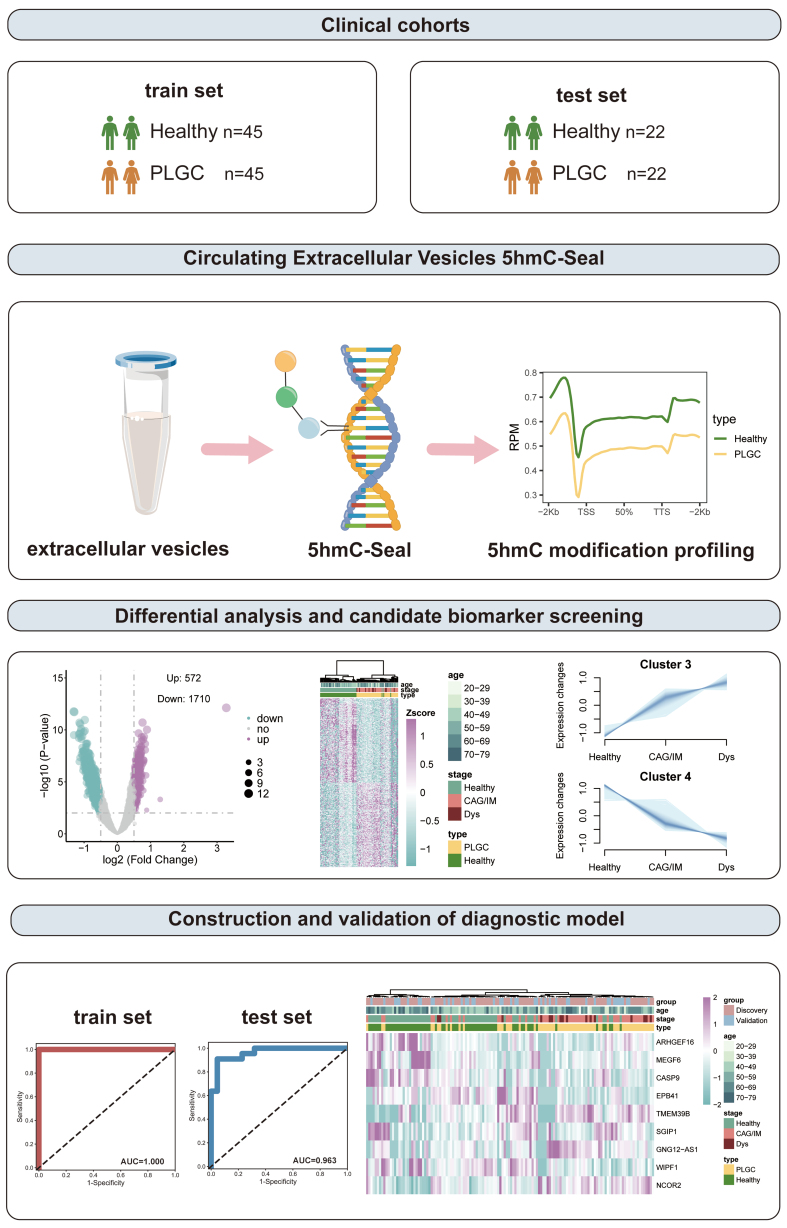
Schematic overview of the study. PLGC: Precancerous lesions of gastric cancer; 5hmC: 5-hydroxymethylcytosine; CAG: chronic atrophic gastritis; IM: intestinal metaplasia; Dys: dysplasia; AUC: area under the curve.

This study collected plasma samples from 67 patients with PLGC and 67 healthy donors. Clinical data were obtained from all samples. Table 1 shows the basic information of patients with PLGC and healthy individuals. There were no significant differences in age and sex. Additionally, we collected operative link on gastritis assessment (OLGA) and operative link on gastric IM assessment (OLGIM) scores for gastric mucosa. We randomly divided all samples into a training set and a test set in a 2:1 ratio for subsequent analysis, utilizing the training set for differential analysis and model construction, and the test set for model validation [Figure 1].

**Table 1:** Demographics and clinical characteristics of the study participants

**Characteristic**	**PLGC**	**Healthy**
*n*	67	67
Age (years)	54.97 ± 12.36	54.58 ± 11.46
Sex		
Male	32 (47.76%)	34 (50.75%)
Female	35 (52.24%)	33 (49.25%)
OLGA		
0	0 (0%)	0 (0.00%)
1	9 (13.43%)	0 (0.00%)
2	30 (44.78%)	0 (0.00%)
3	28 (41.79%)	0 (0.00%)
OLGIM		
0	5 (7.46%)	0 (0.00%)
1	5 (7.46%)	0 (0.00%)
2	22 (32.84%)	0 (0.00%)
3	35 (52.24%)	0 (0.00%)

PLGC: Precancerous lesion of gastric cancer; OLGA: operative link on gastritis assessment; OLGIM: operative link on gastric intestinal metaplasia assessment.

### 5hmC modification profiling and genome-wide distribution in PLGC and healthy individuals

Following isolation, EVs were comprehensively characterized. NTA showed a narrow size distribution centered at ≈ 100 nm for both PLGC and Healthy samples [Supplementary Figure 2A], a morphology confirmed by negative-stain TEM [Supplementary Figure 2B]. Western blotting revealed the canonical EV markers CD9, CD81, CD63, TSG101 and HSP70, while albumin - a proxy for plasma contamination - was virtually undetectable [Supplementary Figure 2C]. Together, these data verify successful isolation of high-purity EVs suitable for downstream analyses.

**Figure 2:**
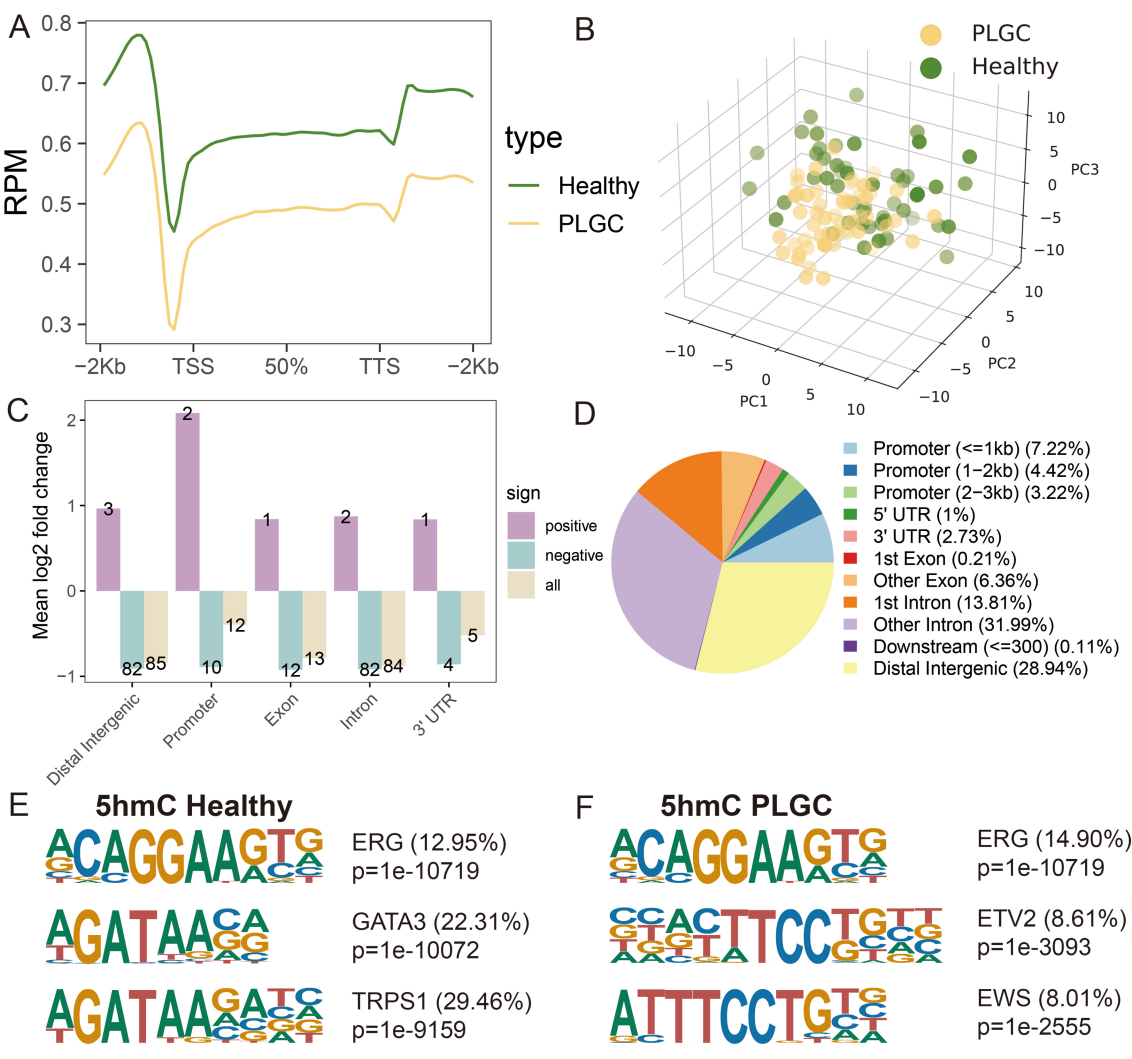
5-hydroxymethylcytosine modification profiling and genome-wide distribution in PLGC and healthy individuals. (A) Distribution of 5hmC in PLGC and healthy individuals at different pathological stages; (B) Principal component analysis results of the two groups; (C) Mean log2 fold change in different genomic regions, including Distal Intergenic, Promoter, Exon, Intron, and 3’UTR; (D) Overall distribution of 5hmC sites in different genomic regions; (E) 5hmC motif analysis results in healthy samples; (F) 5hmC motif analysis results in PLGC samples. PLGC: Precancerous lesion of gastric cancer; 5hmC: 5-hydroxymethylcytosine; TSS: transcription start site; TTS: transcription termination site; PCA: principal component analysis; PC: principal component; UTR: untranslated region; ERG: ETS-related gene; GATA3: GATA binding protein 3; TRPS1: trichorhinophalangeal syndrome type I; ETV2: ETS variant transcription factor 2; EWS: ewing sarcoma gene.

We initially conducted a 5hmC profiling analysis to examine the differences in the 5hmC landscapes of evDNAs between PLGC patients and healthy individuals. We found that the average level of 5hmC within the upstream and downstream 2 kb interval was lower in the PLGC group than in the Healthy group (*p* < 0.05), with the Dys stage showing the lowest levels [Figure 2A]. A PCA-derived distribution plot clearly distinguished PLGC patients from healthy individuals [Figure 2B]. Consistent with previous findings, the 200 highest-ranking 5hmC sites (*P* < 0.05, ranked by |log2 fold change|) were predominantly enriched in distal intergenic and intronic regions [Figure 2C]. Overall, 5hmC sites were mainly enriched in the 1st intron region (13.81%), other introns (31.99%), and distal intergenic regions (28.94%) [Figure 2D].

Additionally, to explore the correlation between 5hmC and potential binding proteins, we performed motif enrichment analysis across the entire genome for 5hmC. In line with prior studies, the E-26 transformation-specific-related gene (ERG) motif (*P* = 1 × 10^-10719^, 12.95%) was the most significantly enriched in both PLGC and healthy evDNAs samples^[38,53]^. In healthy samples, the second and third most enriched motifs were GATA-binding protein 3 (GATA3, *P* = 1 × 10^-10072^, 22.31%) and trichorhinophalangeal syndrome-1 (TRPS1, *P* = 1 × 10^-9159^, 29.46%), respectively [Figure 2E]. In PLGC samples, the second and third most enriched motifs were Ets Variant Transcription Factor 2 (ETV2, *P* = 1 × 10^-3093^, 8.61%) and Ewing sarcoma (EWS, *P* = 1 × 10^-2555^, 8.01%) [Figure 2F]. These transcription factors are expressed in various cell types and are involved in processes such as cell proliferation, differentiation, and apoptosis. These results indicate that the 5hmC profiles of plasma evDNAs in PLGC and healthy individuals have distinct features and hold potential as plasma biomarkers for distinguishing PLGC from healthy individuals.

### Analysis of 5hmC differences and pathway and function annotations

**Figure 3:**
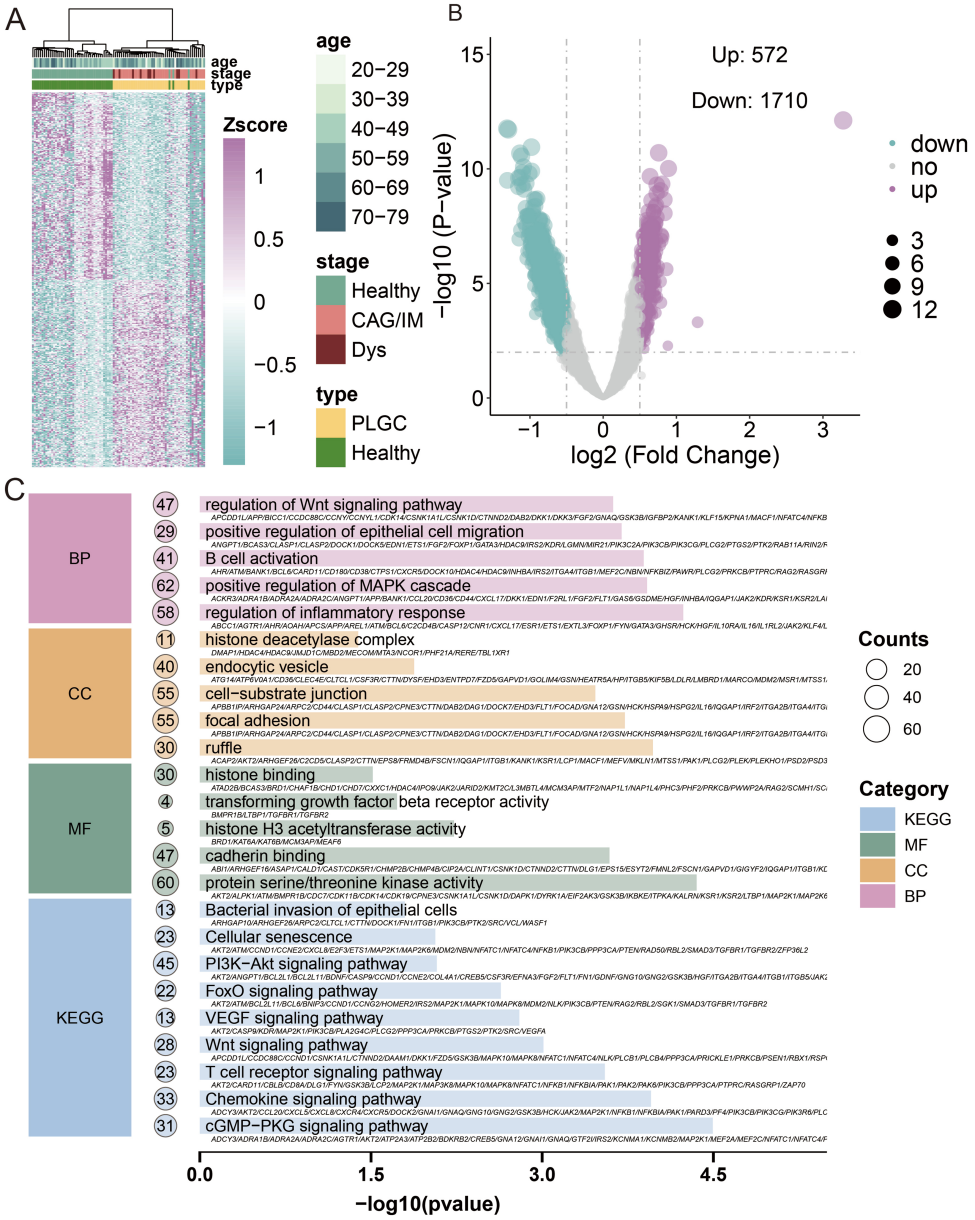
Analysis of 5hmC differences and pathway and function annotations. (A) Heatmap of DhMRs distribution; (B) Volcano plot of DhMRs, with the x-axis representing log2(fold change) and the y-axis representing -log10(*P*-value). Red indicates upregulation, blue indicates downregulation, and gray indicates no significant change; (C) Results of GO and KEGG pathway enrichment analyses. The x-axis shows -log10(*P*-value), and the y-axis lists significantly enriched BP, CC, MF, and KEGG pathways. BP: Biological processes; CC: cellular components; MF: molecular functions; PLGC: precancerous lesions of gastric cancer; CAG: chronic atrophic gastritis; IM: intestinal metaplasia; Dys: dysplasia.

We next performed unsupervised clustering of the top 500 DhMRs (*P* < 0.05; 250 with the highest and 250 with the lowest log2 fold change), which revealed a distinct 5hmC signature separating PLGC patients from healthy controls [Figure 3A and Supplementary Table 1]. For the final DhMR selection used in model construction, we applied more stringent criteria (|log2FoldChange| > 0.5 and *P*-value < 0.01), identifying a total of 2,282 unique DhMRs (572 upregulated and 1,710 downregulated) [Figure 3B]. These DhMRs were then mapped to their corresponding genes to obtain DhMGs, which were subsequently subjected to KEGG pathway and GO enrichment analysis. The results indicated significant enrichment in pathways such as hypoxia-inducible factor 1 (HIF-1) signaling pathway, phosphoinositide 3-kinase (PI3K)-Akt signaling pathway, T cell receptor signaling pathway, Chemokine signaling pathway, Wnt signaling pathway, and Cellular senescence. The GO analysis showed enrichment in biological process (BP) related to positive regulation of epithelial cell migration, regulation of inflammatory response, regulation of Wnt signaling pathway, and B cell activation; CC including focal adhesion, histone deacetylase complex, and cell-substrate junction; and MF (molecular functions) such as transforming growth factor beta receptor activity, cadherin binding, protein serine/threonine kinase activity, and histone H3 acetyltransferase activity [Figure 3C].

Previous studies have shown that PLGC, as a chronic inflammatory disease, is associated with HIF-1, Chemokine, Cellular senescence, and B cell activation^[54-56]^. Additionally, PLGC is closely related to epithelial-mesenchymal transition (EMT) proteins such as beta-catenin and Wnt^[57,58]^. It forms a premalignant microenvironment closely linked to energy metabolism-related pathways such as PI3K-Akt and mitogen-activated protein kinase (MAPK)^[59,60]^. Our findings also showed similar results, indicating that 5hmC in plasma-derived evDNAs is closely associated with the disease, suggesting its potential as a biomarker for monitoring disease progression.

### Identification of DhMRs with trending expression changes and functional annotations

**Figure 4:**
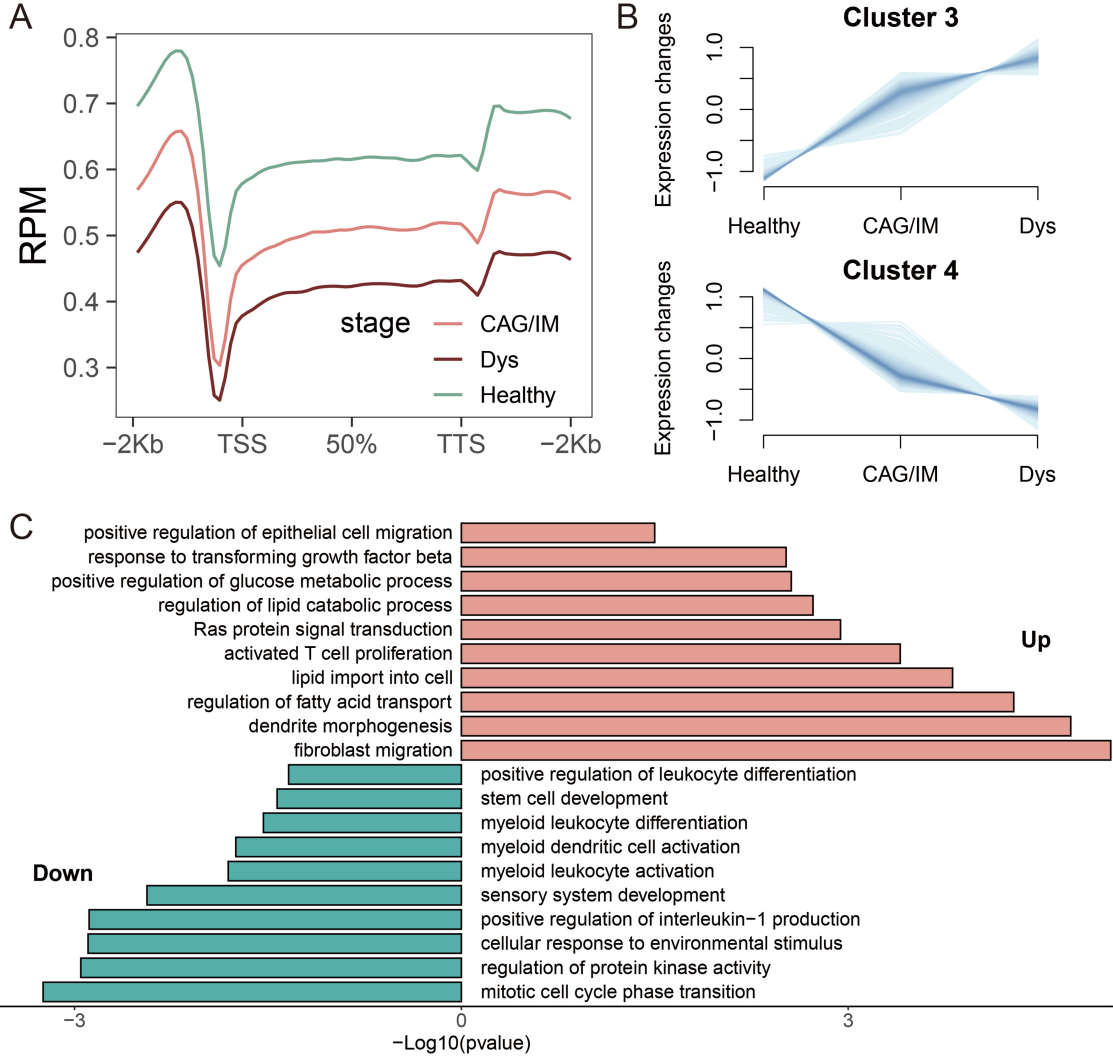
Identification of DhMRs with Trending Expression Changes and Functional Annotations. (A) Distribution of 5hmC in PLGC and healthy individuals at different pathological stages, including CAG/IM, Dys, and Healthy; (B) Clustering analysis to identify clusters with continuous expression changes across healthy, CAG/IM, and Dys states; (C) Enrichment analysis of biological processes for upregulated and downregulated DhMGs. DhMR: Differentially hydroxymethylated region; 5hmC: 5-hydroxymethylcytosine; PLGC: precancerous lesion of gastric cancer; CAG: chronic atrophic gastritis; IM: intestinal metaplasia; Dys: dysplasia; DhMG: differentially hydroxymethylated gene.

According to the Correa cascade, gastric adenocarcinoma is clinically defined as a continuous pathological progression from chronic gastritis, CAG, IM, Dys, to gastric cancer. Based on the results of 5hmC distribution levels, we found that the average levels of 5hmC in the combined CAG/IM group and the Dys group were lower than those in healthy individuals, and they decreased progressively with pathological changes [Figure 4A]. Given the limited number of isolated CAG cases and the biological continuity between CAG and IM, these two stages were analyzed jointly as a single “CAG/IM” group, consistent with recent studies^[61,62]^. The complete DhMR annotations distinguishing Cluster 3 and Cluster 4 are provided in Supplementary Table 2. To further identify DhMRs associated with disease progression, we performed Mfuzz clustering analysis on samples categorized into Healthy, CAG/IM, and Dys groups. This analysis categorized 5hmC sites into four distinct clusters. It is worth noting that Cluster 3 and Cluster 4 exhibited a sustained increasing trend, containing 314 and 985 DhMRs, respectively [Figure 4B]. Subsequent functional enrichment analysis of these continuously upregulated and downregulated DhMRs revealed that upregulated DhMRs were primarily associated with regulation of fatty acid transport, response to transforming growth factor beta, and regulation of lipid catabolic process, while downregulated DhMRs were mainly linked to positive regulation of leukocyte differentiation, myeloid leukocyte differentiation, sensory system development, and stem cell development [Figure 4C].

### Construction and validation of the PLGC diagnostic model

**Figure 5:**
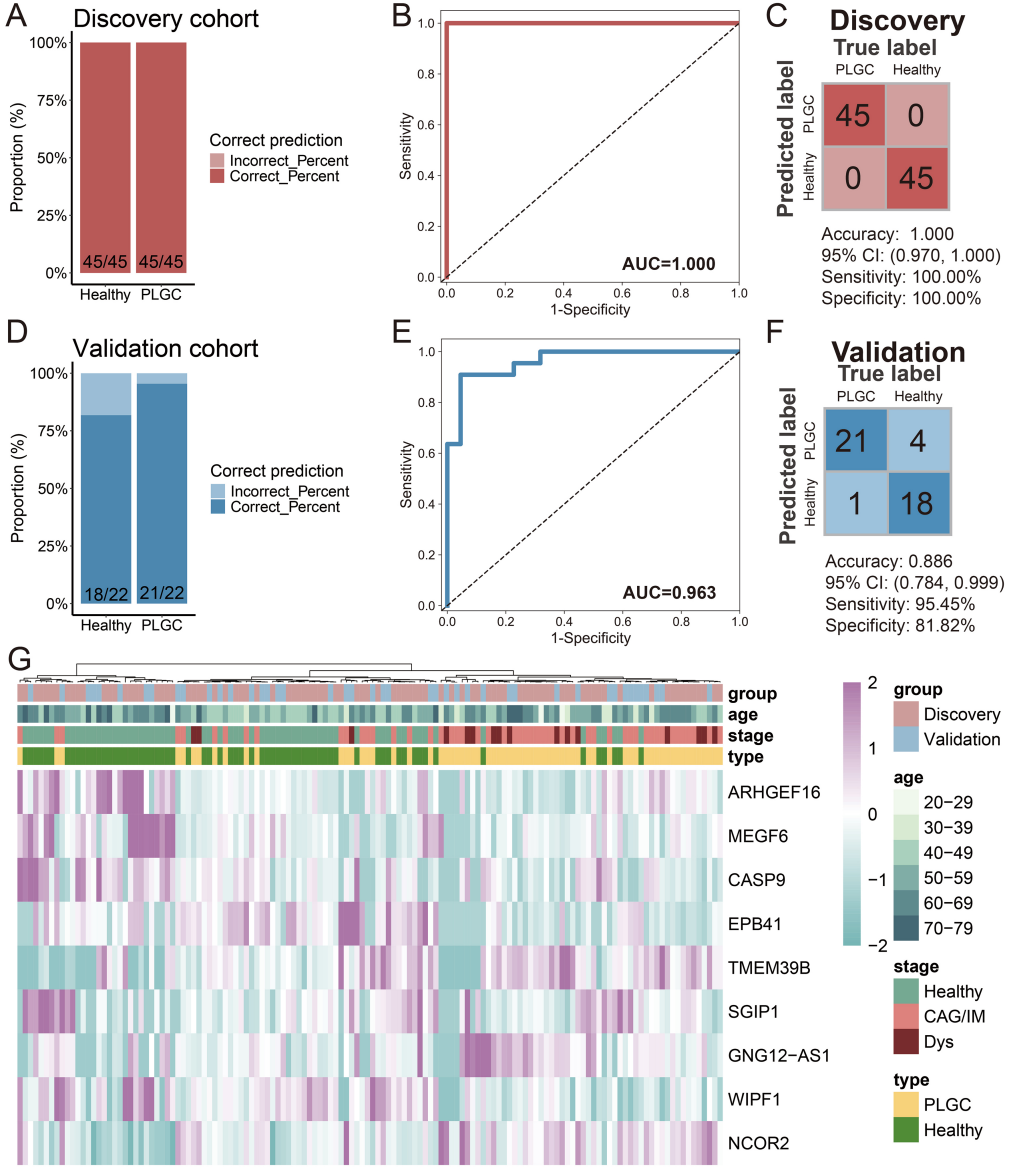
Construction and Validation of the PLGC Diagnostic Model. (A) Stacked bar chart of model prediction results in the training set; (B) ROC curve of the model in the training set; (C) Confusion matrix of the model in the training set; (D) Stacked bar chart of model prediction results in the test set; (E) ROC curve of the model in the test set; (F) Confusion matrix of the model in the test set; (G) Heatmap of DhMGs in the diagnostic model across train and test cohort samples. PLGC: Precancerous lesion of gastric cancer; ROC: receiver operating characteristic; AUC: area under the curve; CAG: chronic atrophic gastritis; IM: intestinal metaplasia; Dys: dysplasia; DhMGs: differentially hydroxymethylated genes.

Next, we conducted feature biomarker screening and diagnostic model construction using 1,664 trend-expressed DhMRs in the training set (90 cases) and validated the model using the test set (44 cases). Initially, we employed linear discriminant analysis, logistic regression, and random forest algorithms for feature selection. We selected the top 30 DhMRs that were chosen by at least two of these algorithms, and ultimately, nine DhMRs [Rho guanine nucleotide exchange factor 16 (ARHGEF16), multiple EGF such as domains 6 (MEGF6), caspase 9 (CASP9), erythrocyte membrane protein band 4.1 (EPB41), transmembrane protein 39B (TMEM39B), SH3GL interacting endocytic adaptor 1 (SGIP1), GNG12, DIRAS3 and WLS antisense RNA 1 (GNG12-AS1), WAS/WASL interacting protein family member 1 (WIPF1), nuclear receptor corepressor 2 (NCOR2)] were incorporated into the diagnostic model [Table 2]. After constructing the diagnostic model, the results showed that the prediction of patients with PLGC in the training set achieved a perfect outcome, with no misdiagnoses among either patients or healthy individuals [Figure 5A]. The AUC value was 1.000 [Figure 5B], with an accuracy of 1.000, sensitivity of 100.00%, and specificity of 100.00% [Figure 5C]. In the test set, the diagnostic model performed well, correctly diagnosing 21 out of 22 PLGC patients and 18 out of 22 healthy individuals [Figure 5D]. The AUC value was 0.963 [Figure 5E], with an accuracy of 0.886, sensitivity of 95.45%, and specificity of 81.82% [Figure 5F]. Unsupervised clustering of the DhMRs in the diagnostic model was further used to generate a heatmap, which confirmed the effectiveness of these DhMRs in distinguishing PLGC patients from healthy individuals [Figure 5G]. These results indicate that DhMRs in evDNAs can serve as diagnostic biomarkers for PLGC, with good diagnostic capability and reliability.

**Table 2:** Characterization-related parameters in the diagnostic model

**Feature**	**95%CI Lower**	**95%CI Upper**
ARHGEF16	-3.81	-3.57
MEGF6	-97.49	-97.29
CASP9	-39.93	-39.57
EPB41	-40.73	-40.41
TMEM39B	117.97	118.33
SGIP1	-182.49	-182.13
GNG12-AS1	149.62	149.94
WIPF1	-32.68	-32.32

### Correlation analysis of DhMGs with clinical indicators

**Figure 6:**
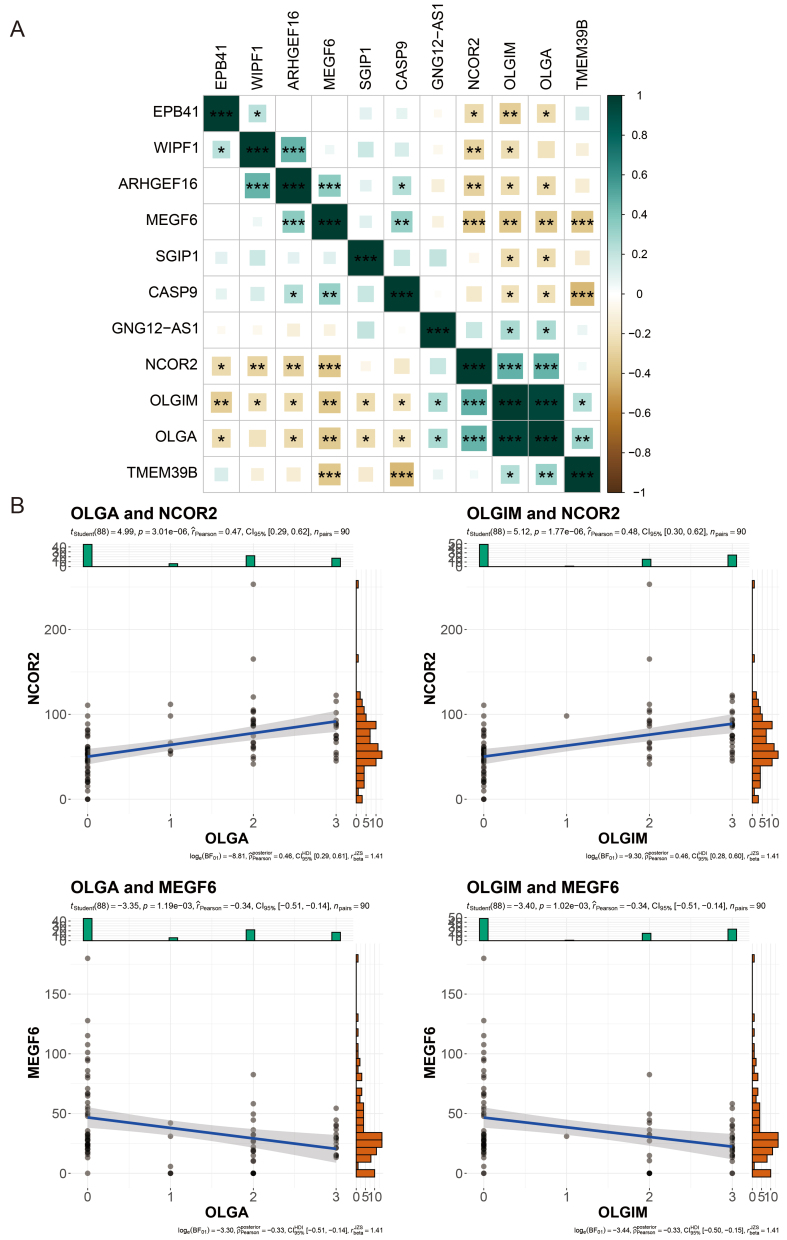
Correlation Analysis of DhMGs with Clinical Indicators. (A) Heatmap of the correlation between different DhMGs and clinical indicators (OLGA and OLGIM); (B) Scatter plots showing the relationships between clinical indicators (OLGA, OLGIM) and the gene expression of DhMGs (NCOR2 and MEGF6) with clinical variables. The y-axis shows the normalized counts of DhMGs per sample, and the x-axis shows the OLGA or OLGIM score. ^*^P < 0.05; ^**^P < 0.01; ^***^P < 0.001. ns: Not significant (Pearson correlation coefficient test). OLGA: Operative link on gastritis assessment; OLGIM: operative link on gastric intestinal metaplasia assessment; DhMGs: differentially hydroxymethylated genes.

Next, we employed correlation analysis to assess the relationship between the nine DhMRs in the diagnostic model and the clinical indicators OLGA and OLGIM, in order to evaluate whether these nine DhMRs are associated with the pathological progression of PLGC. We conducted this assessment using Pearson correlation analysis. The results showed that all nine DhMRs were significantly correlated with both clinical indicators, OLGA and OLGIM. Among them, NCOR2 and MEGF6 exhibited the strongest correlations with OLGA and OLGIM, with *P*-values less than 0.01 [Figure 6A]. Specifically, NCOR2 was positively correlated with OLGA and OLGIM, with correlation coefficients of 0.47 and 0.48, respectively. In contrast, MEGF6 was negatively correlated with OLGA and OLGIM, with correlation coefficients of 0.34 and 0.34, respectively [Figure 6B].

## DISCUSSION

Gastric cancer remains one of the most common cancers globally, with high mortality and morbidity rates due to its weak symptom-disease correlation and the lack of effective curative treatments^[63]^. PLGC represents a crucial stage in the prevention and control of gastric cancer. Effective screening of PLGC, advancing the timing of treatment, and inhibiting pathological progression of the gastric mucosa are of significant importance in reducing the incidence of gastric cancer^[64]^. Currently, the diagnosis of PLGC still relies on pathology and gastroscopy as the gold standard. However, these methods are costly and may lead to complications such as bleeding, perforation, and infection^[10,65]^. Moreover, traditional serum cancer biomarkers, such as carcinoembryonic antigen (CEA), carcinoembryonic antigen (CA72-4), and carbohydrate antigen 19-9 (CA19-9), have proven to be ineffective in screening for precancerous lesions of PLGC^[66,67]^. Therefore, there is an urgent need for novel, non-invasive diagnostic methods that are easy to promote and implement.

Currently, numerous studies have attempted to detect PLGC through biomarkers in blood. For instance, a study utilizing serum proteomics identified a panel of five protein biomarkers and constructed a diagnostic model for PLGC. The model achieved AUC values of 0.763 for low-grade intraepithelial neoplasia and 0.867 for high-grade intraepithelial neoplasia in PLGC^[18]^. Another study combined serum proteomics with mass spectrometry to identify four serum autoantibody biomarkers and incorporated gender into the diagnostic model, resulting in an AUC value of 0.803^[19]^. Additionally, a diagnostic model for PLGC based on circulating microRNA-130b and red cell distribution width demonstrated an AUC value of 0.896^[20]^. However, none of these biomarker-based diagnostic models has achieved high diagnostic efficacy to date. Moreover, most studies have not employed the division of patients into train and test sets for model construction and validation, which may compromise the reliability and generalizability of the models. Therefore, the exploration of new non-invasive diagnostic biomarkers for the screening of PLGC holds significant importance.

Recent studies have indicated a link between PLGC and epigenetics. For instance, the hypermethylation of MIR124-3 and NKX6-1 is associated with the risk of PLGC^[68]^. The m6A modification regulator METTL3 promotes EMT in gastric epithelial cells through the m6A/SNHG7 axis, thereby influencing PLGC^[69]^. Additionally, histone protein deacetylase 6 (HDAC6) reduces forkhead box P3 (FOXP3) via epigenetic modifications, forming a closed loop of HDAC6/FOXP3/hepatocyte nuclear factor 4α (HNF4α) to facilitate the occurrence of IM^[70]^. Recent research has also shown that the 5hmC molecular landscape is closely related to the development of PLGC^[41]^. Therefore, 5hmC holds promise as a novel molecular diagnostic biomarker for the diagnosis of PLGC.

EVs are essential for short- and long-range transport of bioactive molecules, enabling robust intercellular communication^[27,28]^. These EVs can cross endothelial barriers and enter the bloodstream, offering a stable and informative snapshot of disease^[30,31]^. Because they carry lipids, nucleic acids, proteins, glycans and metabolites, EVs have attracted attention as liquid-biopsy targets. They contain gDNA and mitochondrial DNA (mtDNA), so EV-DNA has potential as a biomarker for diverse pathologies, including cancer, tuberculosis, kidney injury and Parkinson’s disease^[71-74]^. The origin of EV-gDNA remains unclear; studies indicate it may derive from chromatid fragments caused by mis-repaired DNA breaks, chromosome mis-segregation, or micronuclei formed by defective nuclear envelopment. Owing to their unstable envelopes, micronuclei eventually rupture, releasing content into the cytosol for engulfment and EV packaging^[75]^. Additionally, both EV release and DNA loading are reported to increase in cancer^[76]^. The 5mC in evDNAs has been reported as a biomarker for gastric cancer^[77]^. Likewise, 5hmC is an important epigenetic mark. Here, using 5hmC-Seal on evDNA, we seek PLGC-specific signatures, build a diagnostic model and assess the utility of EV 5hmC for disease detection.

In this study, we enrolled a total of 67 patients with PLGC and matched them with 67 healthy individuals by age and gender. We performed whole-genome 5hmC sequencing on the evDNAs in their plasma to explore epigenetic biomarkers associated with PLGC. In the process of biomarker selection, we not only identified DhMRs based on traditional differential gene analysis but also employed trend clustering to pinpoint DhMRs associated with the Correa cascade progression, thereby narrowing down the number of candidate biomarkers. Moreover, we adopted a multi-method approach in the feature selection and construction of the diagnostic model. Ultimately, we developed a diagnostic model comprising nine DhMRs (ARHGEF16, MEGF6, CASP9, EPB41, TMEM39B, SGIP1, GNG12-AS1, WIPF1, NCOR2). This model achieved an AUC value of 0.963 and an accuracy of 0.886 in the test set. Although the diagnostic efficiency in the validation set was slightly lower than that in the training set, the performance of our diagnostic model surpassed that of traditional serum markers and previous studies^[18-20]^. Additionally, correlation analysis revealed that all nine biomarkers in the diagnostic model were highly correlated with the clinical pathological scores of OLGA and OLGIM, thereby confirming the reliability of the biomarkers we selected.

As a chronic inflammatory disease, PLGC is associated with oxidative stress and immune activation. Therefore, during the PLGC stage, the expression of pathways such as HIF-1, chemokines, cellular senescence, and B-cell activation can be detected^[54-56]^. Additionally, as a precancerous condition, chronic inflammation that is persistent and recurrent can promote EMT. Studies have found that the expression of the Wnt pathway in PLGC can drive the expression of EMT-related proteins such as β-catenin, thereby facilitating the formation of inflammation-cancer transformation^[57,58]^. Moreover, as inflammation progresses, cellular reprogramming occurs, leading to changes in energy homeostasis. Pathways such as PI3K-Akt and MAPK are also differentially expressed in PLGC^[59,60]^. Our study, through enrichment analysis of DhMRs, identified the expression of the aforementioned pathways. This suggests that the 5hmC in plasma evDNAs has the potential to reflect disease changes and further confirms the reliability of our results.

The nine DhMGs involved in the diagnostic model are primarily associated with functions such as cell adhesion, apoptosis, differentiation, regulation of gene expression, and signal transduction. Among them, CASP9 is closely related to apoptosis. Infection with Helicobacter pylori can promote the activation of CASP9 in B cells, thereby influencing the immune response^[78]^. In the gastric mucosa, trefoil factor 1 (TFF1) can regulate apoptosis by targeting the active form of CASP9^[79]^. WIPF1 is linked to cytoskeleton regulation and cell migration. It can affect the proliferation, invasion, and migration of gastric cancer cells and is associated with the regulation of the PI3K/AKT signaling pathway^[80]^. NCOR2 is a transcriptional corepressor. Research has shown that its methylation status is associated with the density of tumor-infiltrating lymphocytes in gastric cancer^[81]^. Additionally, mutations in NCOR2 are linked to the occurrence of multiple gastric cancers^[82]^.

Although the diagnostic model constructed in this study has demonstrated good diagnostic efficacy, there are still some limitations. First, the samples in this study were derived from PLGC patients and healthy individuals in China. To achieve clinical application in the future, it will be necessary to consider differences between geographic regions and ethnic groups and conduct large-scale studies and validations. In addition, this study also adopted a case-control study design. In subsequent studies, historical longitudinal studies and front-looking trials are crucial for validating and confirming the medical applicability of such methods, ultimately achieving non-invasive detection of PLGC. Finally, the modest sample size precluded subtype-specific modelling; future work will expand the cohort to validate model performance and benchmark it against clinical indicators such as miR-130b, CEA, CA72-4 and CA19-9, refining the 5hmC-based model and yielding additional biological insight.

In conclusion, we have established a non-invasive diagnostic model for PLGC based on the 5hmC landscape in plasma evDNAs. This biomarker panel, consisting of nine 5hmC markers, exhibits high sensitivity and specificity. Our research findings indicate that the 5hmC expression profile is a promising tool for the early detection and accurate diagnosis of PLGC.

## DECLARATIONS

### Availability of data and materials

The data supporting the findings of this study are available from the corresponding author upon reasonable request.

### Copyright

© The Author(s) 2025.
