# Supplementary material for: 5-hydroxymethylcytosine signature in plasma extracellular vesicle DNA as a diagnostic molecular biomarker for precancerous lesions of gastric cancer
Source: Extracell Vesicles Circ Nucl Acids. 2025 Nov 19;6(4):822–42. doi: 10.20517/evcna.2025.76 (PMC12809687; doi:10.20517/evcna.2025.76)
Supplement: Supplementary file 1 [file evcna-6-4-822-SupplementaryMaterials.zip › evcna6076-SupplementaryMaterials/evcna6076-SupplementaryMaterials.pdf]

Supplementary Materials

5-hydroxymethylcytosine signature in plasma extracellular vesicle DNA as a diagnostic molecular biomarker for precancerous lesions of gastric cancer

Haoyu Chen[1,#], Tianyu Gao[1,#], Hangyu Chen[2,#], Lei Zhang[2], Xianglong Chen[3], Maimaitiyasen Duolikun[4], Xiaxuan Li[5], Xuehui Li[2], Long Chen[2,6,7], Han Gao[2], Qi Li[1], Xinyu Hao[8], Pingping Zhou[9], Ningning Ren[10], Jian Lin[2,4,6,7], Yangang Wang[1,10]

[1]School of Graduate, Hebei University of Chinese Medicine, Shijiazhuang 050091, Hebei, China.

[2]Department of Pharmacy, Peking University Third Hospital, Beijing 100191, China.

[3]School of Information and Intelligent Engineering, University of Sanya, Sanya 572022, Hainan, China.

[4]Key Laboratory of Tropical Biological Resources of Ministry of Education, School of Pharmaceutical Sciences, Hainan University, Haikou 570100, Hainan, China.

[5]School of Information and Communication Engineering, Hainan University, Haikou 570228, Hainan, China.

[6]Synthetic and Functional Biomolecules Center, Beijing National Laboratory for Molecular Sciences, Peking University, Beijing 100871, China.

[7]Department of Pharmacy, Peking University Third Hospital Cancer Center, Beijing 100191, China.

[8]Department of Traditional Chinese Medicine, Peking University Third Hospital, Beijing 100191, China

[9]Department of Gastroenterology, Hebei Province Hospital of Chinese Medicine, Shijiazhuang 050011, Hebei, China.

[10]Department of Gastroenterology, Beijing University of Chinese Medicine Third Affiliated Hospital, Beijing 100029, China.

[#]These authors contributed equally to this work.

Correspondence to: Prof. Jian Lin, Department of Pharmacy, Peking University Third Hospital, Beijing 100191, China. E-mail: linjian@pku.edu.cn; Prof. Yangang Wang, School of Graduate, Hebei University of Chinese Medicine, Shijiazhuang 050091, Hebei, China. E-mail: piwei001@163.com

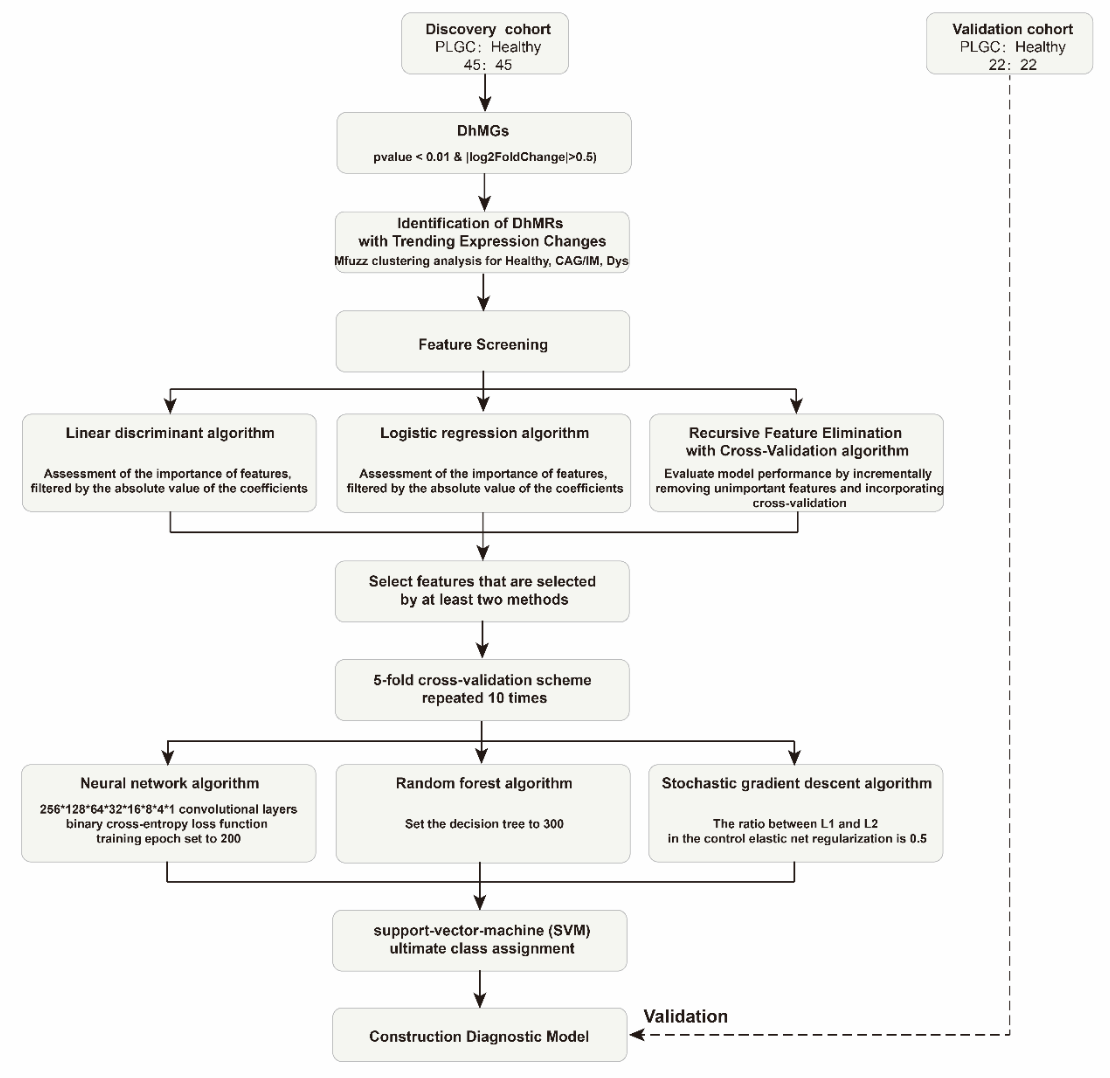

Supplementary Figure 1. Modeling workflow diagram. DhMGs differential hydroxymethylated genes. DhMRs, differential hydroxymethylated regions.

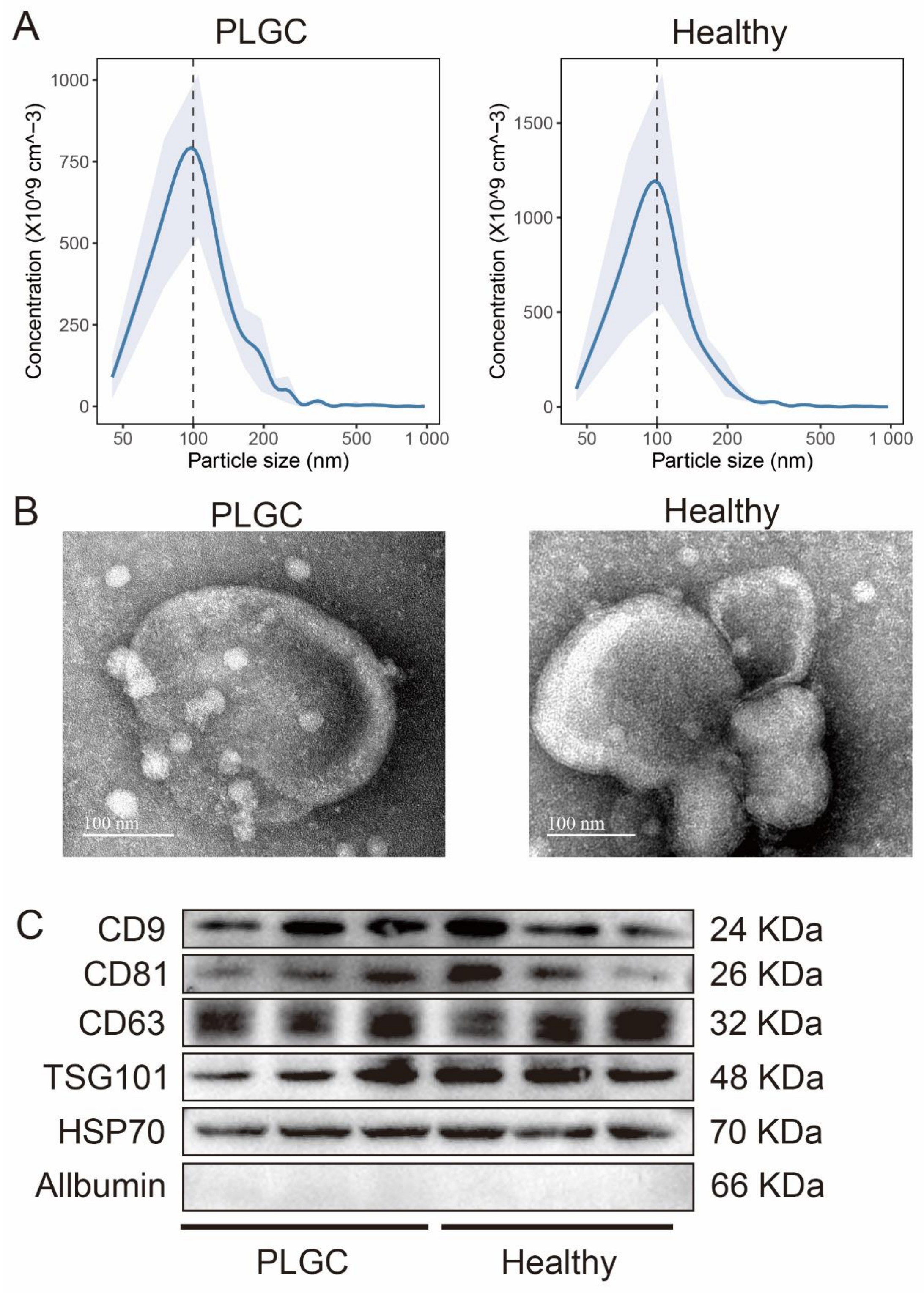

Supplementary Figure 2. EV characterization. (A) Size distribution of EVs derived from PLGC and Healthy samples. The solid blue line indicates the mean diameter determined by NTA; (B) TEM images of EVs isolated from PLGC (left) and Healthy (right) plasma; (C) Western blot characterization of EVs isolated from PLGC and Healthy donors. Positive EV markers: CD9, CD81, CD63, TSG101 and HSP70; negative marker: Albumin.
